# Effect of team quality on offensive playing style and ball possessions development during the 2023 FIFA Women’s World Cup

**DOI:** 10.5114/biolsport.2025.148539

**Published:** 2025-04-14

**Authors:** Iyán Iván-Baragaño, Álvaro Bustamante-Sánchez, Antonio Ardá, Rubén Maneiro

**Affiliations:** 1 Universidad Europea de Madrid, Department of Sport Sciences, Faculty of Medicine, Health and Sports, Spain; 2 Department of Physical and Sport Education, University of A Coruña, A Coruña, Spain; 3 Faculty of Education and Sport Sciences, University of Vigo, Spain

**Keywords:** Women’s soccer, Female football, Performance analysis, Ball possession, Clustering analysis

## Abstract

The aim of this study was to investigate the influence of team quality on offensive strategies related to ball possession in the FIFA Women’s World Cup 2023. Additionally, it sought to examine, through bivariate and multivariate analysis, the influence of the initial offensive intention on the development of ball possessions and the different types of possessions executed in this championship. To achieve this, a nomothetic, punctual, and multidimensional observational study was conducted on 2,346 ball possessions. Three types of analyses were performed: i) contingency table analysis to examine the association between team quality and criteria related to ball possessions, ii) factorial ANOVA to explore the interaction between offensive intention and team quality, and iii) two-way cluster analysis to segment the ball possessions. The results revealed statistically significant differences in 12 criteria associated with the start, development, and outcome of ball possessions, both individually and in combination with initial offensive intention. Furthermore, the two-way cluster analysis identified three types of possessions (Direct Attack, Conservative Attack, and Mixed Approach), with statistically significant differences based on team quality. These results may indicate a shift in elite women’s football offensive strategies, where top teams are able to dominate ball possession and manage offensive strategies in terms of duration and offensive intention.

## INTRODUCTION

Women’s football has experienced significant growth in recent years. By entering the term “female soccer” into the PUBMED database, it becomes evident that in the past six years, nearly the same number of studies (n = 2020) have been published as in the entire previous period (1970–2018, n = 2056). This data reflects the strength of the surge in research around this sport, which has traditionally been far removed from its male counterpart [1, 2].

Although variables of various dimensions have been addressed in women’s football (psychology, physiology…), tactical-strategic aspects have been a significant focus of research, with an emphasis on different performance indicators such as set pieces, possession time, shots on goal, number of goals, or the number of passes completed [3]. It is well known that football is a low-scoring sport compared to others, which is why considering these performance indicators can help assess a team’s tactical performance, not only measured by goals

Regarding these performance indicators, studies focusing on ball possession have gained particular relevance in the scientific literature. Undoubtedly, this performance indicator is one of the most important for achieving goals during competition in female football [4] as it is one of the most efficient mechanisms for building the offensive process. Moreover, significant differences exist between the levels of teams [5, 6]. In this context, scientific literature confirms that the best teams have more possessions when winning than when losing, and they also maintain the ball in the offensive midfield for longer periods [7] with greater passing accuracy [8]. Additionally, there was an observed increase in possession time during the 2023 FIFA World Cup compared to the 2019 edition, specifically by 13.5% [6] highlighting the importance teams place on this variable. Moreover, it has been found that while possessions during the first half of a match generate more attacks, more goals are scored in the second half [9]. Achieving victory often depends on scoring before the opponent [10, 11], which can be facilitated by dynamic ball recovery [12].

Regarding the ball possession zone and intra-team organization, Zubillaga et al. [13] found that player distances increased in depth but decreased in width when possession occurred in regions near either goal. In contrast, when possessions took place in central areas of the field, the opposite was observed: the players’ distribution was significantly wider and less deep. This demonstrates that in women’s football, playing spaces vary depending on the position of the ball. Additionally, possessions that occur in central areas of the field, involving fewer players, are ideal for creating goal-scoring opportunities [14–16].

The method of ball recovery is another variable analyzed in the scientific literature. Dynamic recovery (through ball interception) appears to be a more optimal strategy for improving subsequent offensive actions compared to static recovery (via a regulatory stoppage) [11, 12].

All these findings highlight the intrinsic importance of ball possession in women’s football and how possession can influence various offensive and defensive processes during the game. However, when considering ball possession based on the level of the analyzed teams (top 10 vs. non-top 10 in rankings), the available literature is almost non-existent compared to men’s football. In men’s football, it is well established that the best teams exhibit better and more accurate passing, as well as longer possession times in the opponent’s half [17]. Additionally, studies confirm that top teams commit fewer fouls [18] and manage collective space (width and depth) more effectively than lower-ranked teams [19].

In women’s football, however, studies such as that by O’Donoghue and Beckley [20] analyzed the differences between the best and worst teams in the UEFA EURO 2022, finding that the best teams executed more possessions with 9 or more passes at a slow passing rate (fewer than 1 pass every 3 seconds) compared to lower-ranked teams. These teams also achieved better results in terms of shots on target than possessions with a higher passing rate. Similarly, other studies have analyzed the statistical differences between winning and losing teams in the FWWC19 [21], demonstrating that winning teams had longer possession durations. Likewise, the work of Oliva-Lozano et al. [22] found higher possession levels in winning teams, analyzing the influence of this variable depending on the championship phase. In any case, to the authors’ knowledge, there are no studies in which team level and its interaction with other tactical indicators have been used to examine how the development of possessions is modified in elite women’s football.

Based on the above, the aim of this study was to identify the differences in the context, start, development, and outcome of ball possessions in the FWWC 2023 according to the level of the analyzed teams (Top 10 vs. Non-Top 10). Additionally, it sought to examine, through bivariate and multivariate analysis, the influence of the initial offensive intention on the development of ball possessions and the different types of possessions executed in this championship.

## MATERIALS AND METHODS

### Design

The study was conducted using systematic observational methodology [23] through direct observation of ball possessions during the FIFA Women’s World Cup 2023. Specifically, the design was nomothetic—several units of study corresponding to the participating teams—, punctual— due to its static nature [24], focusing on the analysis of a single championship, albeit with intra-seasonal monitoring of each of the analyzed matches—, and multidimensional—different levels of response analyzed in each of the observable behaviors—, corresponding to the third observational quadrant [24].

### Sample

Using an “all occurrence” observational sampling method [25] all ball possessions from the 16 matches in the Round of 16 and subsequent rounds of the FIFA Women’s World Cup 2023 were analyzed (N = 2,346; Spain = 357; England = 281; Sweden = 257; Australia = 250; Japan = 175; France = 161, Netherlands = 148; Colombia = 134; USA = 96; Denmark = 86; Norway = 75; Nigeria = 69; South Africa = 68; Switzerland = 65; Jamaica = 63; Morocco = 61). Extra time periods were excluded to standardize the number of possessions per match. The inclusion criteria for these actions were set as follows: i) a minimum duration of 4 seconds, and ii) the possession must involve two consecutive touches of the ball, a pass, or a shot (Almeida et al., 2014). These inclusion criteria were defined after an anecdotal record of several randomly selected actions, with the aim of ensuring the inclusion of actions with a collective tactical component.

### Observation and recording instrument

**FIG. 1:**
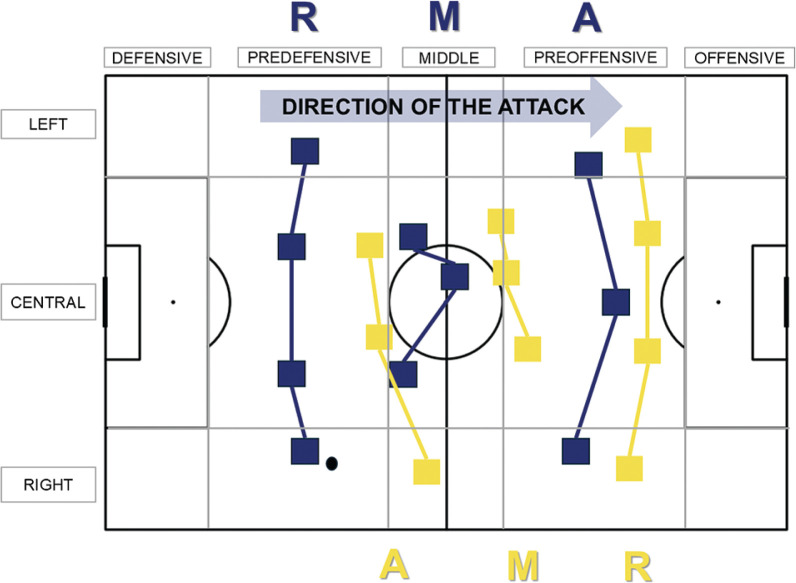
Spatial classification of the field and graphical visualization of the Interaction Context Criteria. Note. The figure is created and adapted from the proposal by Castellano and Hernández-Mendo [28] and Castellano et al. [29]. In this specific example, it can be observed that the ball is in possession of the blue team, with the possessor being a player in the Rear zone, opposing the Advanced zone of the opposing team. This action would be recorded as Interaction Context = RA.

The observation instrument was developed by a panel of experts composed of three researchers with over 30 years of experience in observational methodology (study’s authors). It consisted of 4 dimensions, 18 criteria, and 53 behavioral categories or catalogs. The analyzed criteria included contextual, start, development, and outcome dimensions of the possessions. Team quality was categorized based on the last Ranking FIFA version published before the tournament. Prior to conducting this study, the observation instrument had been used in similar studies with different samples [6, 27]. The dimensions, categories, and operational definitions of each can be found in Table 1. Regarding the spatial criteria, Start Zone (length) and Start Zone (width), the zoning of the playing field can be consulted in Figure 1, along with a graphical description of the Interaction Context criterion, which was categorized and recorded following the proposal by Castellano and Hernández-Mendo [28] and Castellano et al. [29]. Following this proposal, the team’s effective playing space was divided into Rear, Medium, and Advanced zones, as shown in figure 1. This approach allowed the identification of the interaction context based on the opposing zones at the start of ball possession.

**TABLE 1:** Observational instrument: criteria, categories, and operational definition

Criteria	Categories	Operational Definition
**Team Quality**	Top 10	Ranking FIFA Top 10
	No Top10	No Ranking FIFA Top 10

**Match Outcome**	Win	The team observed won the match
	Lose	The team observed lost the match
	Draw	The team observed draw the match

**Time**	1Q	Possession starts between the start of the game and minute 15
	2Q	Possession starts between minute 16 and minute 30
	3Q	Possession starts between minute 31 and the end of the first half
	4Q	Posession starts between the start of the second half and minute 60
	5Q	Possession starts between minute 61 and minute 75
	6Q	Possession starts between minute 76 and the end of thegame

**Match Status**	Winning	The team observed is winning when the action starts
	Drawing	The teams are level when the action starts
	Losing	The team observed is losing when the action starts

**Start Form**	Set Play	Possession begins after a regulatory interruption of the game.
	Transition	Possession begins without a regulatory interruption.

**Start Zone (length)**	Defensive	Possession begins in the defensive area of the pitch
	Predefensive	Possession begins in the predefensive area of the pitch
	Middle	Possession begins in the middle area of the pitch
	Preoffensive	Possession begins in the preoffensive area of the pitch
	Offensive	Possession begins in the offensive area of the pitch

**Start Zone (width)**	Left	Possession starts from the left wing
	Central	Possession starts from the center
	Right	Possession starts from the right wing

**Defensive Organization**	Organised	The opposing team is defensively organised
	Circumstantial	The opposing team is defensively disorganised

**Defensive Positioning**	Low	Opponents positioning is at the back at the start of the action
	Medium	Opponents positioning is midfield at the start of the action
	Advanced	Opponents positioning is forward at the start of the action

**Interaction Context**	MM	Midfield zone vs midfield zone
	RA	Rear zone vs forward zone
	RM	Rear zone vs midfield zone
	AO	Forward zone vs goalkeeper
	AA	Forward zone vs forward zone
	AM	Forward zone vs midfield
	AR	Forward zone vs rear zone
	MA	Midfield zone vs forward zone
	MR	Midfield zone vs rear zone
	PA	Goalkeeper vs forward zone

**Offensive Intention**	Keep	The team observed tries to maintain possession of the ball
	Progress	The team observed tries to progress towards the rival goal

**Defensive Intention**	No pressure	The opposing team shows an intention to defend their goal
	Pressure	The opposing team shows an intention to recover the ball

**MD (seconds)**		Time of possession in own half (in seconds)

**MO (seconds)**		Time of possession in opponent´s half (in seconds)

**Possession Time**		Total time of possession

**Passes**		Number of passes

**Possession Zone**	MD	Most possession in own half
	MO	Most possession in opponent´s half

**Possession Outcome**	Goal	The possession ends with a goal
	Shot	The possession ends with a shot
	Sent to Area	The possession ends with a ball into the penalty area
	No Succes	The possession ends with no success.

Once the observation instrument was completed, the criteria and categories were entered into the Lince Plus v 2.1.0 [30] recording tool, a software for data collection and analysis that enabled the visualization and storage of scenes, as well as the recording and analysis of the actions observed. This open-access software enabled the recording and export of a multievent data type based on the duration parameter [24], which also includes the order and frequency parameters by recording the start and end of each action based on the frame of the imported video. The data exported can be accessed in the repository available with the complete dataset.

### Procedure and reliability

Before recording and coding all actions, the three observers underwent 4 training sessions and familiarization with the observation instrument according to the procedure outlined by Losada & Manolov [25]. Data quality control was performed using Cohen’s kappa coefficient [31] yielding an average value of 0.869 (range .746–.979) from the observer pairs, which was considered excellent [32]. This average represented both the inter-observer and intra-observer reliability after recording a total of 258 ball possessions from two randomly selected matches. To ensure intra-observer consistency in the recording, the researcher responsible for recording the possessions analyzed the initial sample (n = 258) twice leaving three weeks between each observation. The results of Cohen’s Kappa coefficient [31] for each criterion and each pair of observers (intra- and inter-observer) are presented in Table 2.

**TABLE 2:** Intraobserver and interobserver reliability of the observational instrument

Criteria	O1O2	O1O3	O1O4	O2O3	O2O4	O3O4	$\bar{\text{x}}$
**Match Outcome**	0.939	0.913	0.906	0.853	0.860	0.847	**0.886**
**Time**	0.995	0.977	0.986	0.972	0.981	0.963	**0.979**
**Match Status**	0.915	0.981	0.971	0.896	0.886	0.971	**0.936**
**Start Form**	0.839	0.953	0.916	0.864	0.828	0.943	**0.890**
**Start Zone (length)**	0.911	0.947	0.926	0.880	0.848	0.874	**0.897**
**Start Zone (width)**	0.882	0.827	0.876	0.926	0.988	0.926	**0.904**
**Defensive Organization**	0.707	0.907	0.829	0.657	0.613	0.763	**0.746**
**Defensive Positioning**	0.795	0.777	0.789	0.970	0.970	0.818	**0.853**
**Interaction Context**	0.818	0.823	0.813	0.951	0.779	0.723	**0.817**
**Ofensive Intention**	0.872	0.887	0.911	0.793	0.801	0.816	**0.846**
**Defensive Intention**	0.829	0.790	0.839	0.618	0.670	0.632	**0.729**
**Possession Zone**	0.891	0.930	0.961	0.822	0.868	0.892	**0.894**
**Possession Outcome**	0.823	0.809	0.777	0.870	0.830	0.806	**0.819**
** $\bar{\text{x}}$ **	**0.871**	**0.891**	**0.890**	**0.861**	**0.850**	**0.852**	**0.869**

*Note.* O1: Observer 1 – First Recording; O2: Observer 2; O3: Observer 3; O4: Observer 1 – Second Recording

### Statistical analysis

First, an association analysis was conducted using contingency tables to test the hypothesis of dependence between the Top 10 criterion and the other qualitative criteria analyzed. The effect size was calculated using the contingency coefficient, with values considered small (ES = .10), moderate (ES = .30), and large (ES = .50) [33]. For the criteria MD (seconds), MO (seconds), Possession Time, and Passes, differences were tested using the independent samples t-test, justified by the number of possessions in each group. In this case, the effect size was calculated using Cohen’s d and categorized as trivial (ES < .20), small (ES < .60) or moderate (ES < 1.20) [34, 35].

Secondly, a two-way cluster analysis was performed to assess the primary offensive criteria that differentiate the playing style of the teams. The two-way cluster analysis was chosen because it allows for adjustments based on both continuous and categorical criteria considered to be specific descriptors of clusters. This model also provides the feature importance percentage for the clustering classification to better know those aspects that best characterize each group. Normality assumptions were tested using the Kolmogorov-Smirnov test, while homoscedasticity assumptions were checked with the Levene test. Descriptive statistics were reported as mean and standard deviation. After the clustering was completed, differences between clusters based on team rankings were tested using chi-square tests and contingency tables. Additionally, a factorial ANOVA test was used to compare the effects of team ranking (top-10 vs. under top-10), offensive intention (to keep vs. to progress), and the interaction between team ranking and offensive intention. Pairwise comparisons were analyzed using the Bonferroni post hoc test. The significance level for all comparisons was set at p < 0.05. Effect sizes were assessed using eta squared (η^2^) as specified in previous research [36].

The IBM SPSS statistical package version 27.0 for Windows (IBM Corp., Armonk, NY) was used to analyse the data.

## RESULTS

Table 3 presents the results of the association analysis between the FIFA Ranking criterion and the other analyzed criteria. Statistically significant differences were found in the criteria match outcome (p < .001; ES = .485), time (p = .036; ES = .071), match status (p < .001; ES = .148), start zone (length) (p = .031; ES = .067), defensive positioning (p = .004; ES = .069), offensive intention (p < .001; ES = .078), defensive intention (p < .001; ES = .063), MD (seconds) (p < .001; ES = .219), MO (seconds) (p = .002; ES = .118), possession time (p < .001; ES = .245), passes (p < .001; ES = .259), and possession outcome (p = .002; ES = .08).

**TABLE 3:** Bivariate results based on Ranking FIFA

		Top 10 (n = 1550)	No Top 10 (n = 756)	p-value (ES)
**Match Outcome**	Win	778 (50.2%)^*^	157 (19.7%)^**^	**< .001 [.485]**
	Draw	525 (33.9%)^*^	69 (8.7%)^**^	
	Lose	247 (15.9%)^**^	570 (71.6%)^*^	

**Time**	1Q	290 (18.7%)	125 (15.7%)	= **.036 [.071]**
	2Q	250 (16.1%)	115 (14.4%)	
	3Q	266 (17.2%)	152 (19.1%)	
	4Q	268 (17.3%)	117 (14.7%)	
	5Q	231 (14.9%)	133 (16.7%)	
	6Q	245 (15.8%)^**^	154 (19.3%)^*^	

**Match Status**	Winning	455 (29.4%)^*^	67 (8.4%)^**^	**< .001 [.148]**
	Drawing	941 (60.7%)^*^	332 (41.7%)^**^	
	Losing	154 (89.9%)^**^	397 (49.9%)^*^	

**Start Form**	Set Play	503 (32.5%)	256 (32.2%)	= .887 [-]
	Transition	1047 (67.5%)	540 (67.8%)	

**Start Zone (length)**	Defensive	253 (16.3%)	134 (16.8%)	= **.031 [.067]**
	Predefensive	450 (29.0%)	276 (34.7%)	
	Middle	416 (26.8%)	200 (25.1%)	
	Preoffensive	366 (23.6%)^*^	153 (19.2%)^**^	
	Offensive	65 (4.2%)	33 (4.1%)	

**Start Zone (width)**	Left	391 (25.2%)	197 (24.7%)	< .370
	Central	769 (49.6%)	417 (52.4%)	
	Right	390 (25.2%)	182 (22.9%)	

**Defensive Organization**	Organized	14 (1.8%)	782 (98.2%)	= .527
	Circumstantial	22 (1.4%)	1528 (98.6%)	

**Defensive Positioning**	Advanced	555 (35.8%)^**^	324 (40.7%)^*^	= **.004 [.069]**
	Medium	286 (18.5%)	165 (20.7%)	
	Low	709 (45.7%)^*^	307 (38.6%)^**^	

**Interaction Context**	MM	601 (38.8%)	291 (36.6%)	= .553 [-]
	RA	570 (36.8%)	295 (37.1%)	
	RM	37 (2.4%)	18 (2.3%)	
	A0 1 (0.1%)	0 (0.0%)		
	AA	20 (1.3%)	15 (1.9%)	
	AM	14 (0.9%)	8 (1.0%)	
	AR	112 (7.2%)	66 (8.3%)	
	MA	25 (1.6%)	21 (2.6%)	
	MR	13 (0.8%)	3 (0.4%)	
	PA	157 (10.1%)	79 (9.9%)	

**Offensive Intention**	Keep	930 (60.0%)^*^	413 (51.9%)^**^	**< .001 [.078]**
	Progress	620 (40.0%)^**^	383 (48.1%)^*^	

**Defensive Intention**	No Pressure	910 (58.7%)	415 (52.1%)^**^	**< .001 [.063]**
	Pressure	640 (41.3%)	381 (47.9%)^*^	

**MD (seconds)^a^**		8.83 ± 9.99	6.82 ± 7.41	< **.001 [.219]**

**MO (seconds)^a^**		8.08 ± 8.52	7.11 ± 7.58	= **.002 [.118]**

**Possession Time^a^**		16.82 ± 12.97	13.87 ± 10.03	< **.001 [.245]**

**Passes^a^**		4.52 ± 4.07	3.55 ± 3.11	< **.001 [.259]**

**Possession Zone**	MD	786 (50.7%)	412 (51.8%)	.630 [-]
	MO	764 (49.3%)	384 (48.2%)	

**Possession Outcome**	Goal	22 (1.4%)	8 (1.0%)	= **.002 [.08]**
	Shot	113 (7.3%)	53 (6.7%)	
	Sent to Area	256 (16.5%)^*^	87 (10.9%)^**^	
	No Success	1159 (74.8%)	648 (81.4%)	

Note.

a Results shown as mean ± standard deviation.

* More observed than expected values (Z > 1.96);

** Less observed than expected values (Z < 1.96).

**FIG. 2:**
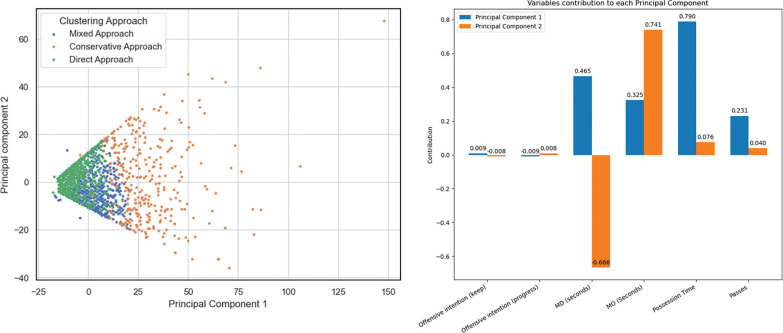
Two-dimensional representation (PCA) of ball possessions based on the clustering group and the influence of each variable on the principal components Note. The percentage of variance explained by the two principal components was 74.25% and 24.67%

Table 4 shows the clustering analysis. We obtained a silhouette index of cohesion and separation of 0.7, which is considered a good cluster quality, and created a cluster membership variable (i.e. “playing style”). The importance of each variable is expressed by its feature importance percentage and ordered from highest to lowest. The offensive intention, the number of passes and the total time were the variables that best discriminated the different playing styles. The two-way cluster analysis table provides the centroids for all the variables that contributed to explaining the model. These values are the average expected behaviour for each playing style. Those variables that did not contribute were not included in the table. Figure 2 illustrates the two-dimensional graphical representation (PCA) of ball possessions and the assignment to each of the clusters.

**TABLE 4:** Results of clustering

Cluster	1	2	3

Size	47.5% (1115)	38.4% (902)	14.0 % (329)
**Variable 1 (Feature importance: 100%)**	Offensive intention: maintain (100%)	Offensive intention: progress (100%)	Offensive intention: maintain (69.3%)

**Variable 2 (Feature importance: 100%)**	Number of passes: 3.74	Number of passes: 2.12	Number of passes: 11.4

**Variable 3 (Feature importance: 100%)**	Total time: 14.04 s	Total time: 9.51 s	Total time: 39.17 s

**Variable 4 (Feature importance: 80%)**	MD time: 8.18 s	MD time: 3.45 s	MD time: 20.92 s

**Variable 5 (Feature importance: 60%)**	MO time: 5.97 s	MO time: 6.08 s	MO time: 18.39 s

*Note*. Numeric values presented as mean value of the cluster; MD time = Ball possession duration (in seconds) in own´s half; MO time = Ball possession duration (in seconds) in opponent´s half.

Taking into account the characteristics of the clusters, three main attacking styles were identified, showing significant differences based on team ranking (p < .001). In this context, Non-Top 10 teams executed 45.1% of their possessions in Cluster 1, 45.2% in Cluster 2, and 9.7% in Cluster 3. In contrast, Top 10 teams executed 48.8%, 35%, and 16.3% of their possessions in the three clusters, respectively. This distribution can be interpreted as follows:

– Cluster 1 (dark blue) (47.5%). Mixed approach: initial offensive intention to maintain the ball possession with an average possession time and number of passes.
– Cluster 2 (dark green) (38.4%) – Direct approach: Progress quickly to find a goal opportunity as fast as possible.
– Cluster 3 (orange) (14%) – Conservative Approach: Maintain possession to look for the best possible goal opportunity.

To finish, Table 5 showed the results of passes and possession time by offensive intention and level. There were differences for the number of passes between top-10 and under top-10 teams. Top-10 teams did more passes with both maintain and progress intentions. There were differences for the number of passes between different offensive intentions. Top-10 and under top-10 teams did more passes when their intention was to maintain the possession. There were also differences for the total possession time between top-10 and under top-10 teams. Top-10 teams had more possession time than under top-10 teams with both maintain and progress intentions. There were also differences for the possession time between different offensive intentions. Top-10 and under top-10 teams had more possession time when their intention was to maintain the possession.

**TABLE 5:** Results of passes and possession time by offensive intention and level

	Keep				Progress			

	Top-10 Level		Under Top-10 Level		Top-10 Lev el		Under Top-10 Level		Intention and Level		

	M	SD	M	SD	M	SD	M	SD	F	p	η^2^
**Number of Passes**	5.43*^‡^	4.11	3.92^‡^	2.99	3.25*	3.62	2.40	2.06	1940.83	< 0.001	0.453

**Total Possession** **Time**	19.35*^‡^	13.17	15.27^‡^	10.36	13.18*	11.46	10.46	6.99	2813.97	< 0.001	0.546

**Own´s half** **Possession Time**	10.89*^‡^	10.06	9.09^‡^	8.64	5.27	8.23	4.44	5.12	1276.00	< 0.001	0.353

**Opponent´s half** **Possession Time**	8.59*	9.30	6.22	7.06	7.93*	7.60	6.05	5.88	1399.31	< 0.001	0.374

*Note*. M: Mean. SD: Standard Deviation. F: Fisher-Snedecor test. η2: Partial eta squared.

* Differences between Top-10/Under Top-10 groups (p < 0.05).

‡ Difference with progress intention (p < 0.05).

We found differences for own´s half possession time between top-10 and under top-10 teams. Top-10 teams had more possession time than under top-10 teams, but only when the goal was to maintain the possession. We did not find significant differences between top-10 and under top-10 own´s half possession time when the intention was to progress. There were also differences for the possession time between different offensive intentions. Top-10 and under top-10 teams had more possession time when their intention was to maintain the possession.

We also found differences for the opponent´s half possession time between top-10 and under top-10 teams. Top-10 teams had more opponent´s half possession time than under top-10 teams with both maintain and progress intentions. We did not find significant differences between top-10 and under top-10 opponent´s half possession time for any of the offensive intentions (both keep and progress).

## DISCUSSION

The present study had a twofold objective. First, it aimed to identify differences in the context, start, development, and outcome of ball possessions in the FWWC 2023 based on the level of the analyzed teams (Top 10 vs. Non-Top 10). The second objective was to examine, through bivariate and multivariate analysis, the influence of the initial offensive intention on the development of ball possessions and the different types of possessions executed in this championship.

Regarding the first objective, the identification of up to 12 criteria showing statistically significant differences between the considered groups indicates that substantial differences existed between higher-ranked and lower-ranked teams in terms of tactical management of ball possession, as well as aspects related to the initiation of possessions. Specifically, the top teams (Top 10) had more ball possessions in the matches they won and drew, as well as at specific moments in the game, such as the start of both the first and second halves. This finding is consistent with previous research [22], reinforcing the idea that top teams aim to impose their style of play on their opponents by using ball dominance through possession to advance towards the opponent’s goal, regardless of the current match status [7]. It is important to note that, except for particular circumstances such as set pieces [37], goals are the result of successful possessions in 60–70% of cases [38].

Differences were also found in the strategic management of space, particularly in the starting zone of ball possessions, with a focus on depth. This variable has been studied in previous research on men’s football [19, 39], and the available results corroborate the idea that top teams more frequently initiate ball possession in key pre-offensive zones, close to the opponent’s goal, similar to patterns observed in men’s football.

Significant differences were also found in the criteria defensive positioning, offensive intention, and defensive intention. Top teams less frequently started from a low tactical position, exhibiting a higher percentage of actions with an offensive intention to maintain possession and gradually build attacks through player passes [40]. Additionally, Top 10 teams demonstrated a greater defensive intention to apply initial pressure on the ball holder. This observation is supported by players’ reports [41] and empirical studies [42].

In direct relation to possession, the statistically significant relationship with possession time (both in own half and in the opponent’s half) aligns with the aforementioned criteria. Top teams have more possession time in women’s football [27] as well as in men’s football [43]. This aspect has been evident in recent years due to the technical improvements experienced by top teams [44]. In the last FWWC23, the two finalist teams averaged a passing accuracy of 84% and 85% [38], four percentage points higher than the team with the highest average in the FWWC19. Finally, the outcome of possession has generally been demonstrated as clearly ineffective, with almost 75% to 80% of possessions ending unsuccessfully, corroborating previous studies conducted during the FWWC2015 [45].

In this regard, we must consider the possibility that, although the offensive level of the teams improves, their defensive performance also improves similarly, potentially leading to a balance in the effectiveness of ball possessions.

Regarding the second objective, a factorial ANOVA test was used to compare the effects of team ranking, offensive intention, and the interaction between both criteria. As observed in Table 5, the results were consistent with those from the first analysis, showing that top teams achieved better scores in terms of the number of passes, total possession time, and possession time in the opponent’s half. Again, these results corroborate previous studies [46] which demonstrated, with moderate and large effect sizes, that the teams that won their matches in the FWWC2019 made a higher number of passes with greater accuracy or a higher number of shots, among other differences, indicating that top teams exhibit more effective strategic management of possessions, both in ball association and time spent, with lower-ranked teams needing to adapt to the tactical scenarios proposed by the higher-ranked teams.

Finally, the results from Table 4 and Figure 2, corresponding to the clustering analysis, allowed for the classification of ball possessions into three complementary groups based on the criteria associated with each type of possession. Each cluster captured different criteria related to the development of possessions, such as offensive intention, total possession time, or number of passes, which can be identified as potential playing styles.

Specifically, Cluster 1, characterized as a mixed approach, was the most prevalent (47.5%, n = 1115), featuring average possession time and number of passes, with an initial intention to maintain the ball. This playing style may be associated with defensive approaches [47], reflecting that top teams manage ball possession defensively when leading in the score. However, further analysis of the interaction of other criteria within this cluster would be necessary. Importantly, no statistically significant differences were observed between Top 10 and Non-Top 10 teams within this cluster, as assessed by the standardized adjusted residuals obtained from the expected and observed values for both types of teams.

On the other hand, Cluster 2 (38.4%, n = 902) was identified with a direct play approach and showed statistically significant differences in the number of possessions between Top 10 and Non-Top 10 teams. Top 10 teams executed approximately 35% of their possessions in this cluster, compared to 45.2% for Non-Top 10 teams. Finally, Cluster 3 was the most conservative offensively, characterized by a distribution in initial offensive intention and a longer possession duration (in fact, the average duration of this cluster can be considered an statistical outlier within the total distribution of the 2,346 analyzed possessions). It is logical to think that this cluster grouped possessions with unusually high durations, which, although not common, represents a substantial percentage of possessions. These possessions mostly have values below the average but include some teams that, in specific situations, exhibit elevated durations and passes, resulting in distributions clearly skewed to the left (e.g., most possessions with a low number of passes but with significant outliers that shift the average to the right). Only 9.7% of the possessions by Non-Top 10 teams were grouped in this cluster, compared to 16.7% for Top 10 teams.

In this sense, the clustering analysis allowed us to observe previously demonstrated aspects. For instance, a lower proportion of passes in Cluster 3 by top teams suggests a lower ability of lower-ranked teams to maintain ball possession [44] and a greater intention to progress quickly towards the opponent’s goal [45]. Moreover, in line with recent studies on women’s football [6, 22, 48, 49], there appears to be a trend shift in offensive strategies in women’s football. Top teams, thanks to improved technical and tactical performance, are capable of controlling ball possession to impose their style of play, moving away from the previous predominance of rapid attacks and counterattacks [12]. This was evidenced when the external load of the players was analyzed in the same championship. In the recent FWWC23, the analysis of physical variables based on the opponent revealed a modification in the players’ external load depending on the opponent’s performance level [50], which may indicate a greater ability to control these parameters. Similarly, we might consider that the results of this study suggest a similar trend at the technical-tactical level, although further research is needed in this regard.

## Limitations and future lines of research

This study presents certain limitations. First, it should be noted that external factors may influence the playing style of national teams, which were not considered in this study. Aspects such as player style (e.g., technical or physical players) can impact a team’s playing model and the offensive approach proposed by the coach. Similarly, studying a single championship does not allow for a high degree of generalizability of the results and, consequently, limits external validity. Lastly, the Team Quality variable does not account for the level of the opponent, a factor that may influence the technical and tactical behavior of the teams.

In future studies, the implementation of multi-championship research could increase the degree of generalizability and, therefore, the applicability of the results. Additionally, the observation of new criteria (e.g., quality of opposition) could better capture the influence of the opposing team on the playing strategies of the analyzed teams.

## CONCLUSIONS

The results of this study demonstrated the existence of significant differences in the start, development, and outcome of ball possessions based on team level, categorized according to the FIFA ranking. Similarly, the interaction between the criteria Offensive Intention and Team Quality showed that Top 10 teams developed ball possessions with longer total duration, both in their own and the opponent’s half, and with a higher number of passes. This was observed in cases where the initial intention was to maintain possession as well as when attempting to progress quickly toward the opponent’s goal.

Finally, possessions were segmented into three major groups (mixed approach, conservative approach, and direct approach), with significant differences observed in the percentage of possessions based on the Team Quality criterion. Top 10 teams displayed a significantly higher percentage of possessions in Cluster 3 (conservative approach), which may indicate a greater ability to dominate the ball and construct positional attacks through collective play in these teams.

## Data availability statement

The data and results that support the findings of this study are openly available in figshare at: https://doi.org/10.6084/m9.figshare.27109414.v1

## Conflict of interest

The authors report there are no competing interests to declare.
